# MLC-LSTM: Exploiting the Spatiotemporal Correlation between Multi-Level Weather Radar Echoes for Echo Sequence Extrapolation

**DOI:** 10.3390/s19183988

**Published:** 2019-09-15

**Authors:** Jinrui Jing, Qian Li, Xuan Peng

**Affiliations:** College of Meteorology and Oceanography, National University of Defense Technology, 60 Shuanglong Road, Nanjing 211101, China; jingjinrui18@nudt.edu.cn (J.J.); pengxuan@nudt.edu.cn (X.P.)

**Keywords:** weather radar sensor, radar echo extrapolation, weather forecasting, deep learning, evolution, spatiotemporal correlation, long short-term memory, adversarial training

## Abstract

Weather radar echo is the data detected by the weather radar sensor and reflects the intensity of meteorological targets. Using the technique of radar echo extrapolation, which is the prediction of future echoes based on historical echo observations, the approaching short-term weather conditions can be forecasted, and warnings can be raised with regard to disastrous weather. Recently, deep learning based extrapolation methods have been proposed and show significant application potential. However, there are two limitations of existing extrapolation methods which should be considered. First, few methods have investigated the impact of the evolutionary process of weather systems on extrapolation accuracy. Second, current deep learning methods usually encounter the problem of blurry echo prediction as extrapolation goes deeper. In this paper, we aim to address the two problems by proposing a Multi-Level Correlation Long Short-Term Memory (MLC-LSTM) and integrate the adversarial training into our approach. The MLC-LSTM can exploit the spatiotemporal correlation between multi-level radar echoes and model their evolution, while the adversarial training can help the model extrapolate realistic and sharp echoes. To train and test our model, we build a real-life multi-level weather radar echoes dataset based on raw CINRAD/SA radar observations provided by the National Meteorological Information Center, China. Extrapolation experiments show that our model can accurately forecast the motion and evolution of an echo while keeping the predicted echo looking realistic and fine-grained. For quantitative evaluation on probability of detection (POD), false alarm rate (FAR), critical success index (CSI), and Heidke skill score (HSS) metrics, our model can reach average scores of 0.6538 POD, 0.2818 FAR, 0.5348 CSI, and 0.6298 HSS, respectively when extrapolating 15 echoes into the future, which outperforms the current state-of-the-art extrapolation methods. Both the qualitative and quantitative experimental results demonstrate the effectiveness of our model, suggesting that it can be effectively applied to operational weather forecasting practice.

## 1. Introduction

The weather radar is one of the important sensors for atmospheric active remote sensing. It transmits a pulse signal into the atmosphere and then receives a part of the signal backscattered by the conglomerate of scatterers (e.g., aerosols, hydrometeors, such as raindrops, snow, etc.) [1]. The received scattering signal, known as weather radar echo, can help forecasters identify and classify weather systems. Beyond this, forecasters can predict the future movement and evolution of weather systems based on radar echo extrapolation, which is the prediction of the appearance, intensity, and distribution of future echoes according to historical echo observations. Thus, radar echo extrapolation has become one of the most fundamental means for short-term weather forecasting and precipitation nowcasting [2,3]. Its significant application value, as well as the difficulty and complexity of the problem, have attracted numerous studies over recent decades.

The earliest research on radar echo extrapolation can be traced back to 1970s [4]. To date, there three categories of traditional extrapolation methods have been developed: centroid tracking, cross-correlation, and optical flow. Centroid tracking methods [5,6,7,8,9] identify the thunderstorm echo cells and track their movement. However, they are limited to the extrapolation of severe convective systems. In comparison, cross-correlation methods [10,11,12,13] take sub-regions of the echo as the extrapolation target and predict their movements. Although cross-correlation methods can deal with stratiform precipitation systems that are gently varied, the prediction of severe convective systems which are fast-moving and evolving rapidly is difficult due to increasing incorrect calculations of motion vectors. Optical flow methods [14,15] calculate the flow field of the echo and extrapolate the radar echo by solving the optical flow constraint equation with additional constraints, such as the Horn–Schunck global smoothing constraint [16] and the Lucas–Kanade local constraint [17]. Optical flow methods assume that there are only motion changes in continuous echoes, regardless of the changes in echo intensity in the time sequence, which is not coincident with the reality that echoes change with both position and intensity through time. Overall, these three methods only trace back a few steps for previous echoes and their capability of modeling non-linear motion patterns is weak. Thus, they have inherent limitations, such as short timeliness and low precision.

In recent years, some researchers have applied deep learning [18], which can effectively learn representations of data with multiple levels of abstraction and has achieved significant progress in computer vision, to deal with radar echo extrapolation. Compared to conventional radar echo extrapolation methods, deep learning based methods possess greater modeling capacity as they can fit sophisticated designed neural networks by learning from large-scale historical radar echo datasets. Generally, there are two main types of deep learning models, the convolutional neural network (CNN) [19,20,21] and the recurrent neural network (RNN) [22,23,24], while the CNN specializes in extracting spatial features from a single frame, the RNN is more suitable for capturing temporal correlation from sequential data. For the extrapolation methods based on CNN, Klein et al. (2015) [25] proposed a dynamic convolutional layer applying a dynamically varied convolution kernel on the last input echo frame to extrapolate, where the kernel is generated based on input, but it is limited to extrapolating only one echo frame at a time. Shi et al. (2018) [26] combined a recurrent dynamic sub-network and a probability prediction layer to form their model, where the recurrent dynamic sub-network is a CNN incorporated with a recurrent structure. For the RNN models, Shi et al. (2015) [27] first defined the radar echo extrapolation problem as a spatiotemporal sequence prediction problem, then proposed the Convolutional Long Short-Term Memory (ConvLSTM), in which the dot products in the fully connected LSTM [28] are substituted by the convolution operations, to better capture spatiotemporal correlations from radar echo sequences. In addition, they built an encoding-forecasting structure with ConvLSTM as a basic building block for sequence-to-sequence multistep prediction. Wang et al. (2017) [29] extended the ConvLSTM with an additional spatiotemporal memory integrated with its origin memory cell and conveyed across the network vertically; they showed that this would be conducive to learn spatial and temporal representations simultaneously. Shi et al. (2017) [30] considered that the local weather systems sometimes have motion patterns, such as rotation and scaling, and the location-invariant kernel used by the convolution operation may not be sufficient to model them. Thus a structure generating network was used to dynamically generate a local connection structure between hidden states for state-to-state transition. To improve the image quality of the extrapolated echoes, Tran and Song (2019) [31] employed some of the visual image quality assessment metrics including the structural similarity index measure (SSIM) and multi-scale SSIM to train their model.

Although the above deep learning based extrapolation methods have exhibited a remarkable improvement in extrapolation performance compared with traditional methods as they can forecast more complicated temporal motion patterns and some spatial deformations caused by the motion, there are still two limitations which should be considered. For the first, in reality, the variations of weather systems are more than only advection motion, weather systems will simultaneously experience an evolutionary process from formation to dissipation, which also affects the extrapolation accuracy but has been rarely investigated by the existing methods. And for the second, the extrapolated echo of existing deep learning methods tends to be increasingly blurry as the extrapolation goes deeper, which may due to the widely used mean square error (MSE) or mean absolute error (MAE) loss functions [32,33] as they lead to averaging all potential predictions and lose echo details. In our work, these two limitations are considered, and a deep learning model is constructed to predict the echo evolution and extrapolate echo more accurately. First, motivated by the physical characteristics of weather systems’ evolutionary process, a variant of the RNN unit called Multi-level Correlation LSTM (MLC-LSTM) is proposed to exploit the spatiotemporal correlation between multi-level radar echoes and model the echo’s spatiotemporal evolution. To be specific, there are usually abundant vertical, horizontal, and diagonal atmospheric motions existing in weather systems [34], which drive the evolution and development of weather systems (which can be viewed as 3D entities) and cause them to have a strong spatiotemporal correlation between different height levels. Thus, it makes sense that the MLC-LSTM can take multi-level echoes as input and exploit their spatiotemporal correlation for extrapolation that will fit the physical conditions more adequately and contribute to performing prediction of evolution more effectively. Second, to address the problem of blurry echo prediction, the recent success of generative adversarial network (GAN) [35,36,37] has inspired us to integrate adversarial training into our approach, which is training a generator and a discriminator in an alternative way to lead the generated data distribution to match the real data distribution. Thus, the generated data could be sharp and realistic. We first construct an encoder–predictor architecture based on the MLC-LSTM for end-to-end radar echo extrapolation to act as the generator, then design a CNN structure as the discriminator. They are trained with both the image loss and the adversarial loss to lead the extrapolation echo results to be more fine-grained and realistic.

For model training and testing, we have built a real-life multi-level radar echoes dataset. Through the extrapolation experiments conducted on this dataset, the effectiveness of the different components of our model has been verified first, then compared with other state-of-the-art extrapolation methods. The results show that our model can extrapolate radar echo more effectively and accurately and that it has important application value in weather forecasting practice.

The rest of the paper is organized as follows; the proposed model is described in detail in Section 2. The dataset, experiments settings, evaluation metrics, effectiveness analysis of model components, qualitative and quantitative evaluation results are presented in Section 3. The work of this paper is summarized, and an outlook of future work is given in Section 4.

## 2. Model

In this section, the formulation of the radar echo extrapolation problem and an overview of our proposed model is given first in Section 2.1. Then the details about the MLC-LSTM and the encoder–predictor architecture are presented, respectively, in Section 2.2 and Section 2.3. At last, the discriminator and the loss functions used in this paper, including the image loss and the adversarial loss, are introduced in Section 2.4.

### 2.1. Model Overview

Weather radar echo extrapolation is the prediction of the appearance, intensity, and distribution of future echo sequences according to historical echo observations. It can also be formulated as a spatiotemporal sequence prediction problem, that is, given a length M historical echo sequence $\chi_{t-M+1:t}^{L1:Ln}$ as input, where L1:Ln denote 1:*n* altitude levels, and each echo image can be represented as a tensor $\chi \in {\mathbb{R}}^{w\times h\times c}$, w, h and c are width, height and number of channels, respectively, the goal of the extrapolation is to predict the most-likely length N future echo sequence ${\hat{\chi }}_{t+1:t+N}^{L1:Ln}$ as

(1) $${\hat{\chi }}_{t+1:t+N}^{L1:Ln}=\underset{\chi_{t+1:t+N}^{L1:Ln}}{\arg\max}p(\chi_{t+1:t+N}^{L1:Ln}|\chi_{t-M+1:t}^{L1:Ln}).$$

Unlike most current methods using only a single-level (n=1) echo to extrapolate the same level echo, our model utilizes three-level (n=3) echoes to extrapolate the middle-level echo by sufficiently exploiting the spatiotemporal correlation between them. The three altitude levels chosen in this paper were 2, 2.5, and 3 km since the representative precipitation echo usually abundantly exists between 2 and 3 km altitude, and the middle-level is 2.5 km. The length of the input sequence M and the extrapolation sequence N were set to 10 and 15, respectively.

**Figure 1:**
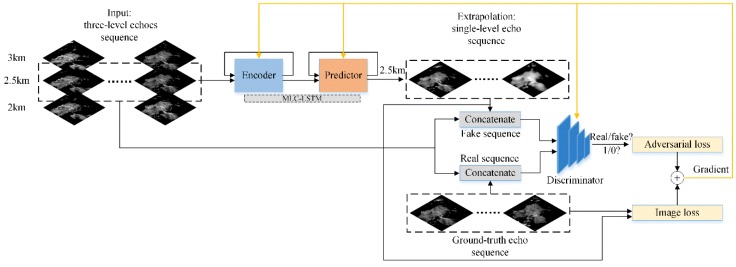
Model overview.

As illustrated in Figure 1, our model was composed of an encoder–predictor architecture and a discriminator, and it was trained with a combination of the adversarial loss and image loss. The encoder–predictor architecture was an RNN structure using the MLC-LSTM as the basic building unit and acted as the generator. The encoder recurrently processed the input echo sequence and encoded them into hidden representations, which contain information about the appearance of echo components and how they were moving and evolving. Then the predictor decoded these representations and generated the extrapolation echo sequence recurrently. The discriminator took the concatenation of the input echo sequence and the extrapolation echo sequence (or the ground-truth echo sequence) as input, outputted a logic probability of whether the sequence would be fake or real (between 0 and 1). The discriminator was a CNN structure consisted of consecutive convolution layers and nonlinear activation function. The adversarial loss and image loss were used jointly for gradient computation, and parameters optimization, where the adversarial loss was calculated based on the probability scalars output by the discriminator and the image loss was derived between the extrapolation echo sequence and the ground-truth echo sequence. The MLC-LSTM, encoder–predictor architecture, discriminator architecture, and loss functions will be described in the following sections.

### 2.2. MLC-LSTM

**Figure 2:**
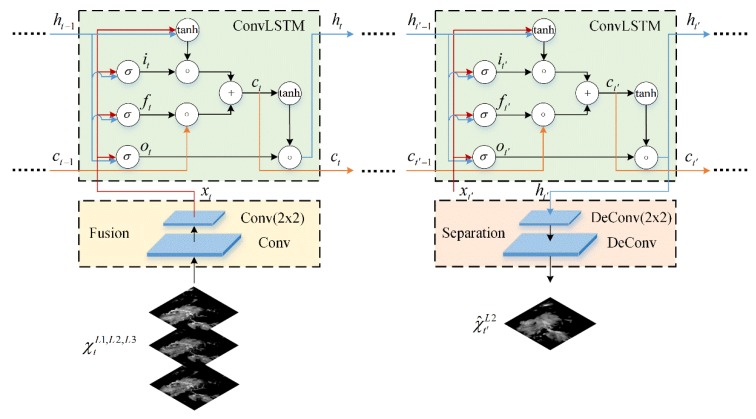
The architecture of the Multi-Level Correlation Long Short-Term Memory (MLC-LSTM).

For a weather system which is in the evolutionary process, there is usually plenty of atmospheric motions inside it which drives its development. While the horizontal motions mainly lead the advection of the weather system, the vertical motions have been regarded as one of the most important driving factors for the evolution. For example, for a convective storm in the dissipation stage, its heavy precipitation center will drop quickly under the control of the downdraft, leading the intensity of high-level precipitation to weaken rapidly and dissipate gradually. Therefore, when considering the radar echo extrapolation, the weather system should be treated as a 3D entity and its spatiotemporal correlation between different levels should be captured for modeling and predicting the evolution more accurately. Motivated by this, we propose the Multi-level Correlation LSTM (MLC-LSTM) to exploit the spatiotemporal correlation between multi-level radar echoes and model their evolution. As shown in Figure 2, the MLC-LSTM consists of the fusion module, ConvLSTM, and separation module.

The fusion module converts the input three-level echoes into feature space and fuses the echo features separated at different levels together. It models the redistribution of echo intensity which is caused by the spatiotemporal atmospheric movements at one timestep. We implement the fusion module with two convolutional layers, the first convolutional layer with stride 1 × 1 is used to map the input echoes from the image space to feature space, and the second layer with stride 2 × 2 is applied to integrate features from different levels. Both of the two layers are followed by the rectified linear unit (ReLU) activation function.

The ConvLSTM is a variant of the LSTM proposed by Shi et al. (2015) [27], which is an effective and fundamental spatiotemporal recurrent structure for spatiotemporal modeling. Here, we use it to take the fused features as input and model the spatiotemporal echo evolutionary process. It works by keeping a memory flow inside and updating the hidden state according to the values of its internal three sigmoid gates: the input gate, forget gate, and output gate. When new input arrives, the input gate controls how much of the new information from the external input will be added to the memory cell, the forget gate controls which previous information will be forgotten from the memory cell, and the output gate controls which cell information will be propagated to the new state. The update equations of the ConvLSTM are as follows:

(2) $$i_{t}=\sigma (W_{xi}*x_{t}+W_{hi}*h_{t-1}+b_{i}),$$

(3) $$f_{t}=\sigma (W_{xf}*x_{t}+W_{hf}*h_{t-1}+b_{f}),$$

(4) $$c_{t}=f_{t}\circ c_{t-1}+i_{t}\circ \tanh(W_{xc}*x_{t}+W_{hc}*h_{t-1}+b_{c}),$$

(5) $$o_{t}=\sigma (W_{xo}*x_{t}+W_{ho}*h_{t-1}+b_{o}),$$

(6) $$h_{t}=o_{t}\circ \tanh(c_{t}),$$

where σ is the sigmoid activation function, ∗ and ∘ denote the convolutional operator and the Hadamard product, respectively. Input $x_{t}$, memory cell $c_{t}$, hidden state $h_{t}$, input gate $i_{t}$, forget gate $f_{t}$, and output gate $o_{t}$ are both 3D tensors. Weights W and biases b are both learning parameters.

The separation module separates the features of the extrapolation level from the state output of ConvLSTM and transforms it back into image space at each extrapolation timestep. It has a symmetrical architecture to the fusion module, with the convolutional layers having been replaced with the deconvolution layers [38]. The first deconvolution layer with stride 2 × 2 selects out the feature maps and the second with stride 1 × 1 transforms the selected features to echo image. As with the fusion module, they are also followed by ReLU activation.

### 2.3. Encoder–Predictor Architecture

**Figure 3:**
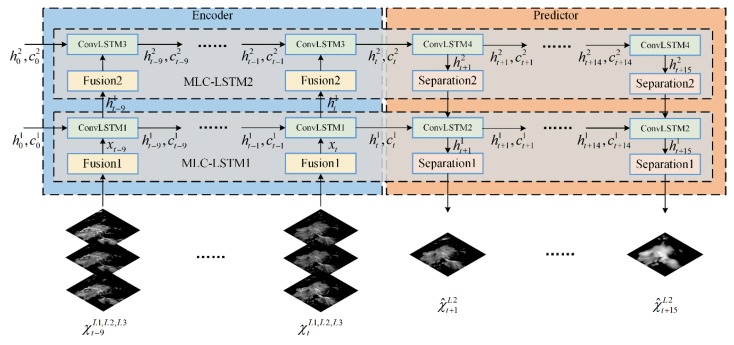
The schematic of the encoder–predictor architecture.

The MLC-LSTM proposed in Section 2.2 can be adopted for modeling the spatiotemporal echo evolution. By stacking it, we construct an encoder–predictor architecture for end-to-end extrapolating echo sequence ${\hat{\chi }}_{t+1:t+15}^{L2}$ given the input echo sequence $\chi_{t-9:t}^{L1,L2,L3}$, as shown in Figure 3.

The encoder recurrently encodes the input echo sequence and converts them into hidden representations. It models the echo evolution in feature space and captures the most critical features which can be used for extrapolation and are encoded into the hidden representations, such as the appearance of the echo components (cell echo or layered echo), their motion and evolution. The predictor decodes the hidden representations and recurrently speculates out the future echo sequence. It makes a reasonable prediction of echo motion and evolution based on the encoded representations to obtain the final extrapolation echo sequence. This encoding–predicting process can be formulated as

(7) $$[h_{t}^{1:k},c_{t}^{1:k}]=f_{encoder}(\chi_{t-9:t}^{L1.L2.L3},h_{0}^{1:k},c_{0}^{1:k}|\theta_{e}),$$

(8) $${\hat{\chi }}_{t+1:t+15}^{L2}=f_{predictor}(h_{t}^{1:k},c_{t}^{1:k}|\theta_{p}),$$

where $h_{0}^{1:k}$ and $c_{0}^{1:k}$ are the initial states and memory cells of the encoder and are initialized with zero tensors. $h_{t}^{1:k}$ are state outputs of the encoder and represent the multi-scale spatial features (appearance) of the echo components, where 1:k denotes the index of the MLC-LSTM, and k is the stack number of the MLC-LSTM. $c_{t}^{1:k}$ are the cell outputs of the encoder, which memorizes the multi-scale temporal variation features (motion and evolution) of echo components. $h_{t}^{1:k}$ and $c_{t}^{1:k}$ constitute the hidden representations encoded by the encoder and also act as the initial states of the predictor. $\theta_{e}$ and $\theta_{p}$ are the learning parameters (weights and biases) of the encoder and predictor, respectively.

In this paper, we stack two MLC-LSTMs (k=2) to form the encoder–predictor architecture, which is a balanced choice between the memory-consuming and the modeling capacity. And to enhance the model generalization ability and increase the extrapolation accuracy, the dropout [39] is added on the ConvLSTM of the first MLC-LSTM in the predictor with a dropout rate of 0.3. In addition, we adopt a minor change for the second MLC-LSTM, that is removing the first convolutional layer from the fusion module and the second deconvolution layer from the separation module, for the reason that the high-level of the network does not need a mapping between the feature space and the image space. The parameter details about the encoder–predictor architecture, including each layer’s input size, number of channels, convolution (deconvolution) kernel size, and convolution (deconvolution) stride, are provided in Table 1.

### 2.4. Loss Functions

For deep learning methods, loss functions are used to calculate the gradient of parameters and optimize the model. However, typical losses, such as MAE or MSE, used by existing extrapolation methods will result in blurry extrapolation results, since they lead the model to generate a medial data distribution of all plausible distributions, and thus the echo details may be lost. To address this problem and extrapolate sharp and realistic, we adopted the generative adversarial training in our approach, which is training a generator and a discriminator to compete with each other, where the discriminator aims to distinguish the generated data from real, and the generator is trying to trick the discriminator into judging the generated data as real. In our model, the encoder–predictor architecture constructed in Section 2.3 acts as a generator and was trained to minimize a combination of the image loss and adversarial loss as

(9) $$L_{gen}=\lambda_{img}L_{img}+\lambda L_{adv},$$

where $\lambda_{img}$ and $\lambda_{adv}$ are the weights corresponding to the image loss and adversarial loss, respectively, the image loss $L_{img}$ is the sum of the MAE and MSE as

(10) $$L_{img}=\frac{1}{N}\sum_{i=1}^{N}\left(\left|\chi_{i}^{L2}-{\hat{\chi }}_{i}^{L2}\right|+{\left(\chi_{i}^{L2}-{\hat{\chi }}_{i}^{L2}\right)}^{2}\right),$$

and the adversarial loss $L_{adv}$ is defined as

(11) $$L_{adv}=L_{bce}\left(D\left(\left[\chi_{t-9:t}^{L2},G\left(\chi_{t-9:t}^{L1,L2,L3}\right)\right]\right),1\right),$$

where $G\left(\chi_{t-9:t}^{L1,L2,L3}\right)={\hat{\chi }}_{t+1:t+15}^{L2}$ denotes the generator, [.,.] denotes concatenation operator along the depth dimension. D is the discriminator that outputs a logic probability of whether the input is from reality (real) or generated by the generator (fake). $L_{bce}$ is the binary cross-entropy loss defined as

(12) $$L_{bce}\left(x,\hat{x}\right)=-\hat{x}\log\left(x\right)-\left(1-\hat{x}\right)\log\left(1-x\right),$$

where x and $\hat{x}$ are logit (between 0 and 1) and label (whether 0 or 1), respectively.

**Figure 4:**
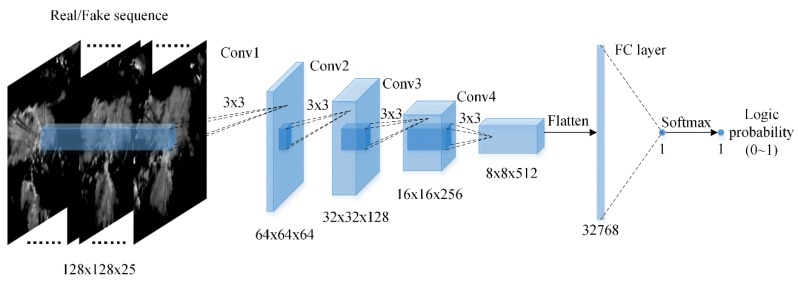
The schematic of the discriminator.

In this paper, we implemented the discriminator D with a CNN structure, as illustrated in Figure 4. It consists of 4 convolutional layers and 1 fully-connected layer, the number of channels of each convolutional layer is 64, 128, 256, and 512, both of the convolutional layers use a 3 × 3 size kernel with a 2 × 2 stride and are activated by the ReLU activation function. For the fully-connected layer, it outputs a single scalar which will be passed through the softmax function to obtain the final logic probability.

While the training objective of the generator G is to make the discriminator D believe that the fake sequence $\left[\chi_{t-9:t}^{L2},G\left(\chi_{t-9:t}^{L1,L2,L3}\right)\right]$ is real, the training objective of the discriminator D is to correctly judge the real sequence $\chi_{t-9:t+15}^{L2}$ as real and the fake sequence as fake. It can be defined as

(13) $$L_{dis}=L_{bce}\left(D\left(\chi_{t-9:t+15}^{L2}\right),1\right)+L_{bce}\left(D\left(\left[\chi_{t-9:t}^{L2},G\left(\chi_{t-9:t}^{L1,L2,L3}\right)\right]\right),0\right).$$

To make the discriminator D and generator G reach a nash equilibrium, which means that the generator G can extrapolate echoes as real as the ground-truth and the discriminator D finds it hard to distinguish them, we perform the training of the discriminator D and generator G alternately with different updating rates. The detailed adversarial training strategy will be described in Section 3.2.

## 3. Experiments and Results

In this section, we have conducted several experiments to verify the effectiveness of our model. In Section 3.1, the construct steps and details of the real-life multi-level radar echoes dataset are introduced. In Section 3.2, the settings of the experiments, including the hyperparameters, adversarial training strategy, and evaluation metrics, are given. In Section 3.3, the effectiveness of different components of our model has been validated. In Section 3.4, experimental results are compared with the state-of-the-art methods and analyzed. In Section 3.5, the performance of the model is evaluated. All the experiments in this paper are implemented using Python, MATLAB, and Tensorflow [40] and conducted on 4 RTX 2080Ti GPUs and 1 Intel Xeon Gold 5118 CPU.

### 3.1. Dataset

Since our model aims at exploiting the spatiotemporal correlation between multi-level radar echoes for extrapolation, we have constructed a real-life multi-level radar echoes dataset. The type of the radar sensor we chose is the CINRAD/SA Doppler Weather Radar [41], which works in the VCP21 detection mode and has a 6-min interval of volume scanning and 9 detection elevations (from 0.5° to 19.5°). The raw radar echo dataset we used was provided by the National Meteorological Information Center, China, which contained data detected and collected by Hangzhou, Nanjing, Xiamen, Changsha, and Fuzhou stations from 2016 to 2017. Considering that rainy days usually have a more effective precipitation echo for model training and validation, a total of 307 rainy days’ data were selected based on the historical daily precipitation observations to construct the final dataset

For data pre-processing, we first interpolated the raw radar echo data into cartesian coordinate to obtain a multi-level constant altitude plan position indicator (CAPPI) images [42]. Since the valid detection radius of the radar was about 240 km on the 2 to 3 km altitudes, the central 480 × 480 (480 × 480 km^2^) region of echo images was cropped and remained. Then, they were resized to 128 × 128 size with bilinear interpolation to be more suitable for model training and test. In addition, the reflectivity factor values of echo images were clipped to be between 0 and 75 dBZ and then normalized into gray-level pixel value which was between 0 and 1. The clutter has also been suppressed.

In our work, 10 historical three-level echo images were input, and the subsequent 15 future echo images on the middle-level were output as extrapolation results. Thus, a sliding window with length 25 and stride 3 was applied on each rainy day’s data to divide them into echo image sequences. A total of 12,144 sequences were obtained and randomly split into a training set of 8508 sequences, a validation set of 1200 sequences, and a test set of 2436 sequences. In the experiments, the training set was used for training the deep learning model, the validation set was used for judging when to adopt early-stopping and adjusting the model hyper-parameters, such as learning rate, kernel size, and dropout rate. All the comparison and evaluation experiments were conducted on the test set.

### 3.2. Experiments Settings

During the training of the MLC-LSTM, we set the weight of image loss $\lambda_{img}$ and adversarial loss $\lambda_{adv}$ to 1 and 0.02, respectively, to make sure that the two sub-loss were both located on the same scale of magnitude. All the neural network weights were initialized with a Xavier initializer [43], and all the biases were initialized to 0. Both of the generator and the discriminator were optimized by the Adam optimizer [44] with momentum $\beta_{1}=0.9$, $\beta_{2}=0.999$, and initial learning rate 0.0001. The adversarial training was launched starting from the discriminator and then the generator. The updating ratio for the generator and discriminator was set as 2:1, which means that the generator was updated 2 steps per updating step of the discriminator as we found that the discriminator usually converges faster than the generator and that the updating ratio can contribute to stabilizing the adversarial training. Since it was hard to decide when to stop training by visualizing the fluctuant training loss, we performed the training for 20000 to 50000 iterations and chose the stopping point when the model performed best on the validation set. The batch size of the training was set to 4.

For quantitative evaluation metrics, we adopted the probability of detection (POD), false alarm rate (FAR), critical success index (CSI) [45], and Heidke skill score (HSS) [46], structural similarity index measure (SSIM) [47], and peak signal to noise ratio (PSNR) [48] in this paper. The SSIM and PSNR are two image-level similarity metrics used widely in the computer vision field, and a higher value denotes a higher similarity. The POD, FAR, CSI, and HSS are commonly used metrics for evaluating the quality of precipitation nowcasting, where the POD represents the ratio of successful predictions to the total number of events, the FAR represents the proportion of incorrect predictions to all predictions, and the CSI and HSS are more comprehensive metrics as they take into consideration both the successful and incorrect predictions. A larger score of the POD, CSI, HSS, and a lower score of the FAR means that the nowcasting quality is better.

To calculate these metrics, we first mapped the pixel values of ground-truth echo and extrapolation echo back to reflectivity factors, then converted the reflectivity factor to rainfall rate using the Z-R relationship as

(14) Z=10loga+10blogR,

where Z(dBZ) denotes the reflectivity factor of radar echo, R(mm/h) is the rainfall rate and a, b are two constants set to 58.53 and 1.56, respectively, according to usual experience.

After that, the ground-truth echo and extrapolation echo were transformed into two 0/1 matrices at a threshold of 0.5 mm/h rainfall rate (the threshold indicating raining or not raining) and the hits $n_{h}$ (ground-truth = 1, extrapolation = 1), misses $n_{m}$ (ground-truth = 1, extrapolation = 0), false alarms $n_{f}$ (ground-truth = 0, extrapolation = 1), and correct rejections $n_{c}$ (ground-truth = 0, extrapolation = 0) were counted. Then, the POD, FAR, CSI, and HSS can be calculated by

(15) $$POD=\frac{n_{h}}{n_{h}+n_{m}},$$

(16) $$FAR=\frac{n_{f}}{n_{h}+n_{f}},$$

(17) $$CSI=\frac{n_{h}}{n_{h}+n_{m}+n_{f}},$$

(18) $$HSS=2\frac{n_{h}n_{c}-n_{m}n_{f}}{\left(n_{h}+n_{m}\right)\left(n_{m}+n_{c}\right)+\left(n_{h}+n_{f}\right)\left(n_{f}+n_{c}\right)},$$

where the POD, FAR, CSI, and HSS are ranges between 0 and 1.

### 3.3. Effectiveness Validation

In this section, we conduct experiments to verify the effectiveness of the different components of our model, including the effectiveness of the model architecture, dropout, and adversarial training strategy, see Section 3.3.1, Section 3.3.2 and Section 3.3.3 for detailed results.

#### 3.3.1. The Effectiveness of Model Architecture

In this paper, we stacked two MLC-LSTMs to form our model architecture. To prove it can balance the memory consumption and the modeling capability, we compared it with two model variants, MLC-LSTM with only one layer and MLC-LSTM with three layers stacked. For the one-layer model variant, it removed the second MLC-LSTM. For the three layers model variant, the additional third MLC-LSTM doubled the number of channels to 256 and had the same 3 × 3 size kernel.

**Figure 5:**
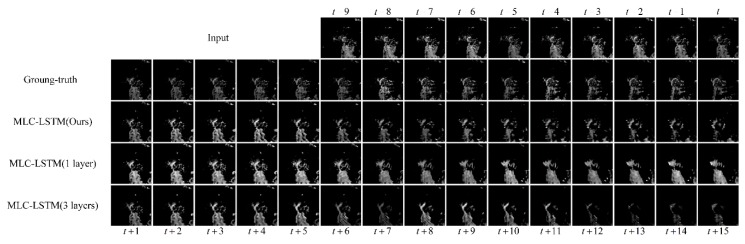
The extrapolation results for the echo at Hangzhou, China, 8 August 2016, 23:07 UTC. The last three rows show the results obtained by our model, MLC-LSTM with one layer, and MLC-LSTM with three layers, respectively.

The echo at Hangzhou, China, 8 August 2016, 23:07 UTC was chosen as a sample for extrapolation. The extrapolation results predicted by the three models are shown in Figure 5. In the ground-truth, the echo located in the southeast was continuously moving to the southwest. Meanwhile, the main part of the echo was gradually separating and dissipating. It can be seen that our model predicted this process accurately, although, in the later stage, the shape of the extrapolated echoes was not fully consistent with the ground-truth. The echo motion and dissipation have been well modeled. Compared with our model, the other two model variants did not predict the echo motion as accurately. For the dissipation process, the MLS-LSTM with only one layer had little ability to predict it. The extrapolated echoes seem not changed. The MLC-LSTM with three layers stacked predicted the dissipation roughly and excessively. It only shrank the whole echo cell and cut the echo contents, not considering that the echo shape had dispersed and changed, which was also not in line with the ground-truth. These differences, we think, may be related to the modeling capability, the two model variants cannot do well as they have a limited and excess modeling capability, respectively, while our model possesses a moderate modeling capability and thus, extrapolates the echo appropriately.

The evaluation results of three models on POD, FAR, CSI, HSS, SSIM, and PSNR metrics are given in Table 2. Our model achieved almost the best scores, except for the POD, which was obtained by the one-layer model variant. This can be explained reasonably, as shown in Figure 5, the MLC-LSTM with a single layer was poor at predicting the dissipation and tends to predict the echo with more incorrect contents. Thus, its POD was higher than the others and FAR was also the highest. In addition, a performance evaluation of our model was also performed, which will be described in Section 3.5. Totally, our model did not consume much memory and simultaneously kept a decent modeling capability.

#### 3.3.2. The Effectiveness of Dropout

**Figure 6:**
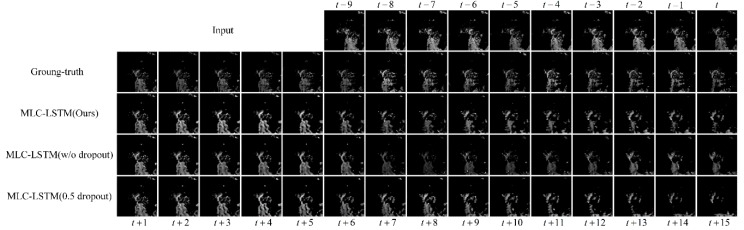
The extrapolation results for the echo at Hangzhou, China, 8 August 2016, 23:07 UTC. The last three rows show the results obtained by our model, MLC-LSTM without the dropout and MLC-LSTM with 0.5 rate dropout respectively.

To verify the effectiveness of dropout we used in this paper, we compared our model with the MLC-LSTM without using the dropout and MLC-LSTM with 0.5 rate dropout. For the same echo sample as Section 3.3.1, the extrapolation results obtained by the three models are shown in Figure 6.

From Figure 6, we can see both of the three models capture the echo motion and predict a satisfactory echo in the first half extrapolation stage (from t + 1 to t + 10), but during the second half (from t + 10 to t + 15), the MLC-LSTM without dropout and MLC-LSTM with 0.5 rate dropout did not predict the dissipation process as good as our model. For the MLC-LSTM without dropout, its predicted echo has a thicker main body than others. This is probably because it has a weak generalization ability so that it makes a maximization hypothesis and tends to predict more potential but incorrect echo contents. For the MLC-LSTM with 0.5 dropout rate, its extrapolation of the echo is more dispersive in distribution and even vanished. This might be because it adopts a relative larger dropout rate, and there are fewer neural connects activated, which diminishes the modeling capability. Overall, our model MLC-LSTM with a 0.3 dropout rate, ensures both the generalization ability and the modeling capability, thus performs better against other models. 

The quantitative evaluation results, as shown in Table 3, indicate that our model also does almost the best on both six metrics quantitatively, which corroborates its effectiveness, too. In addition, the MLC-LSTM without dropout achieves the highest FAR among the three models, which is consistent with the fact that it is inclined to predict more incorrect echo contents.

#### 3.3.3. The Effectiveness of Adversarial Training Strategy

**Figure 7:**
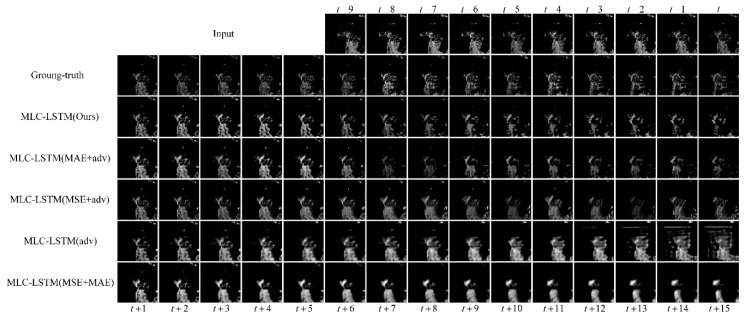
The extrapolation results for echo at Hangzhou, China, 8 August 2016, 23:07 UTC. The last five rows show the results obtained by MLC-LSTM trained with different loss schemes, respectively.

In this section, we aim to verify the validity of our adversarial training strategy. Considering the loss function, in our work, a combination of the image loss, including the MSE and MAE, with the adversarial loss is chosen to avoid the blurry prediction problem and predict realistic echo. Here for comparison, we have also tried another four training loss schemes: training with the MAE and adversarial loss (MAE + adv), MSE and adversarial loss (MSE + adv), adversarial loss only (adv), and image loss only (MSE + MAE). The extrapolation results of the model trained with different loss schemes are shown in Figure 7.

As illustrated in Figure 7, training the MLC-LSTM with MSE + MAE generated a quite blurry prediction of the echo, since it is only guided by optimizing the averaging difference it is hard for the model to generate complicated real echo distribution. The MLC-LSTM trained with the adversarial loss only also has an inferior performance. The generated echoes were less realistic and contained some checkboard artifacts. This may be due to the adversarial loss not being sufficient to constrain the generated data distribution matching the ground-truth since there are many plausible distributions. For the MLC-LSTM trained with the MAE + adv, it predicted the echo with an approximately correct shape and contour but did not contain much texture detail. In contrast, the MLC-LSTM trained with MSE + adv was good at rendering texture details but failed to maintain the echo shape. This difference might be caused by the fact that the MAE and MSE are sensitive to the shape and texture, respectively. Our model trained with the MAE, MSE, and adversarial loss, takes both the echo shape and texture into consideration and generated more realistic extrapolation results.

The quantitative evaluation results of five training loss schemes are given in Table 4. Training with only the adversarial loss obtained the worst performance on six metrics. Training with MSE + MAE achieved the best score of FAR but did not perform well on POD, as it predicted fewer and blurry echo contents. Our training loss scheme obtained a comprehensive best performance, with the highest score of CSI, HSS, and SSIM and the second score of POD, FAR, and PSNR.

**Figure 8:**
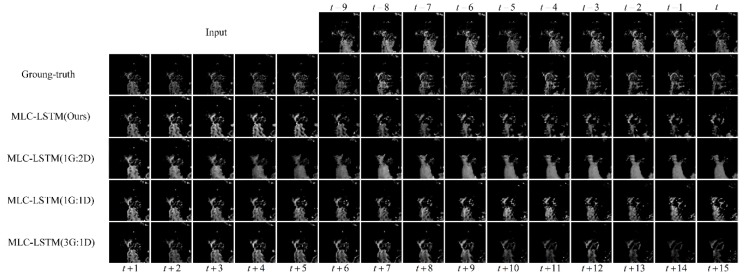
The extrapolation results for echo at Hangzhou, China, 8 August 2016, 23:07 UTC. The last four rows show the results obtained by MLC-LSTM trained with different updating ratios.

Another experiment was carried out to verify the effectiveness of training the generator and discriminator with different updating rates. In this paper, we adopted an updating ratio of 2:1 for the generator and discriminator. To analyze how the updating ratio would affect the model performance, we changed it to 1:2, 1:1, and 3:1, respectively. The extrapolation results and quantitative evaluation results of the model trained with different updating ratios are reported in Figure 8 and Table 5.

It can be seen from Figure 8 that the higher the updating rate of the generator to the discriminator (from 1G:2D to 3G:1D), the fewer echo contents were predicted. The MLC-LSTM trained with updating ratios of 1:2 and 1:1 generated more echo contents than the ground-truth while the 3:1 updating ratio produced less than the ground-truth, and our 2:1 updating ratio was just enough. From the evaluation results shown in Table 5, it also indicates that the POD had a negative correlation with the updating ratio and the FAR had a positive correlation, which was consistent with the predicted echo contents decreasing as the updating ratio increased. The 2:1 updating ratio used in this paper was the most suitable for our model.

Moreover, in our model, the number of parameters of the generator and discriminator were 2,601,601 and 1,590,273, respectively, which was closest to the ratio of 2:1. Therefore, we conclude that a proper updating ratio of the generator and discriminator should be aligned with the ratio of the number of their parameters.

### 3.4. Comparison Experiments

In this section, we conducted comparison experiments to evaluate the effectiveness of our whole model using the best setting described above. The model was compared with two typical traditional extrapolation methods, TREC [10] and Optical flow [17], and two state-of-the-art deep learning methods, ConvLSTM [27] and TrajGRU [30]. In addition, to make a fair comparison and demonstrate the effectiveness of exploiting the spatiotemporal correlation between three-level radar echoes sufficiently, we also compared the model with the ConvLSTM which takes three-level echoes as input and the MLC-LSTM which only receives one-level echo as input. They are denote as ConvLSTM (three-level input) and MLC-LSTM (one-level input).

**Figure 9:**
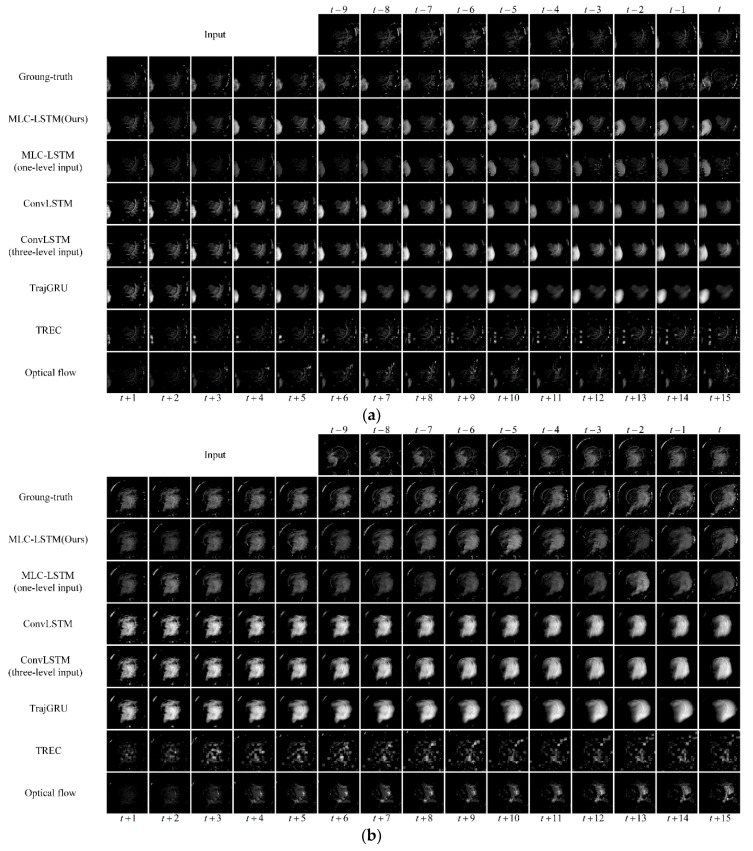

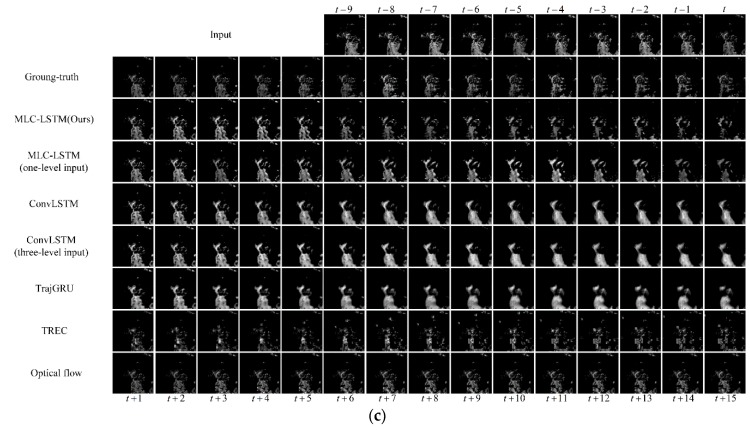
Three extrapolation samples predicted by seven models. (**a**) An echo advection motion process at Nanjing, China, 6 September 2016, 10:20 UTC; (**b**) An echo formation process at Nanjing, China, 6 September 2016, 13:54 UTC; (**c**) An echo dissipation process at Hangzhou, China, 8 August 2016, 23:07 UTC.

The extrapolation samples are shown in Figure 9, including an echo advection motion process at Nanjing, China, 6 September 2016, 10:20 UTC, an echo formation process at Nanjing, China, 6 September 2016, 13:54 UTC, and an echo dissipation process at Hangzhou, China, 8 August 2016, 23:07 UTC. For the two traditional extrapolation methods TREC and Optical flow, the extrapolated echo the shape was hard to maintain, and each patch of the echo dispersed sharply as time went by. The echo formation and dissipation were also barely predicted. This happens as their modeling ability is limited only extrapolating the echo using the motion vectors field, which basically cannot predict the echo evolution. Even for calculating a relative effective motion vector field, additional constraints and complex parameter settings are usually required. Thus, the TREC and Optical flow find it difficult to provide accurate predictions for actual nowcasting practice. For the deep learning models, they were generally superior to the traditional models in both extrapolations of the echo motion, formation, and dissipation, but it can be noticed that after the first few steps of extrapolation (usually 5 to 8 steps), the ConvLSTM and TrajGRU encountered the problem of blurry echo prediction. The extrapolated echo became homogenized, and the echo details were missing. Only in the prediction by the MLC-LSTM was this problem avoided, which can be attributed to the adopted adversarial training. Considering the exploitation of the spatiotemporal correlation, when the ConvLSTM also took the three-level echoes as input, the prediction results remained almost the same as the original ConvLSTM, and the extrapolation performance was not promoted much. When the MLC-LSTM only receives one-level input, the ability to predict the echo evolution reduced. For example, in Figure 9b, its extrapolated echo shape was not consistent with the ground-truth. The MLC-LSTM with three-level echoes input predicts the echo much closer to the ground-truth. Therefore, it demonstrates that our model can exploit the spatiotemporal correlation between three-level radar echoes more effectively and use it to assist in predicting the echo evolution.

**Figure 10:**
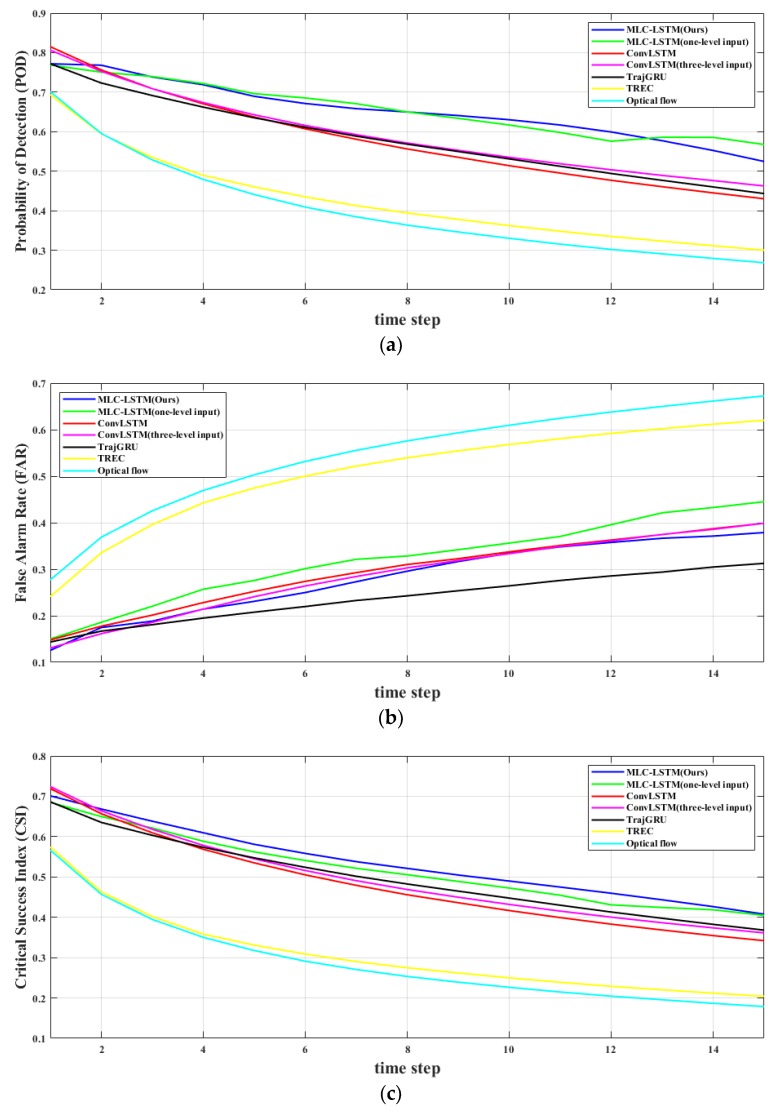

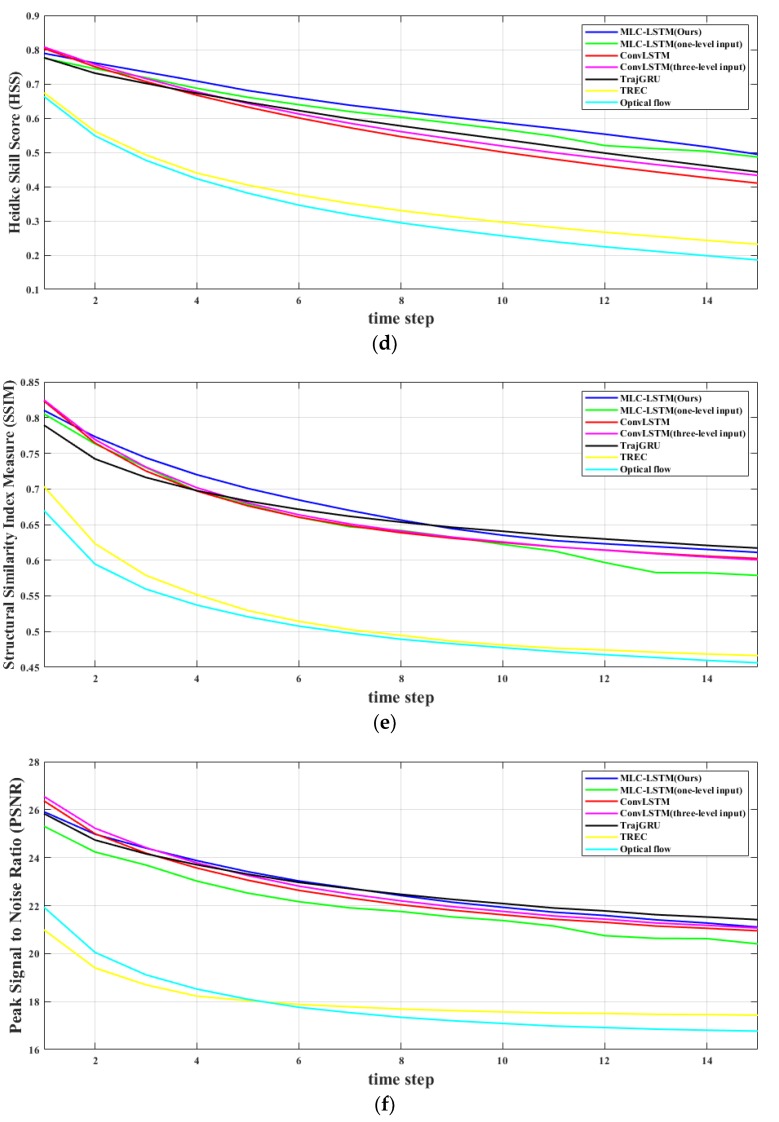
Frame-wise comparison results of seven model on six metrics. (**a**) Probability of detection (POD) changing curve; (**b**) false alarm rate (FAR) changing curve; (**c**) critical success index (CSI) changing curve; (**d**) Heidke skill score (HSS) changing curve; (**e**) structural similarity index measure (SSIM) changing curve; (**f**) peak signal to noise ratio (PSNR) changing curve.

The quantitative evaluation results on extrapolating echo for 0.5, 1, and 1.5 h, and frame-wise comparison results of all models are illustrated in Table 6 and Figure 10, respectively. The two traditional methods, TREC and Optical flow, achieved the lowest performance on all metrics. The ConvLSTM and TrajGRU perform well on FAR. This might simply be because they tend to predict less and more concentrated echo contents. For POD, CSI, and HSS, all the deep learning models perform approximately the same for short-term forecasting (0.5 h). However, when the extrapolation carried forward deeper, our model MLC-LSTM outperformed the ConvLSTM and TrajGRU, which is aligned with the ConvLSTM and TrajGRU suffering from the blurry prediction problem while the MLC-LSTM maintains a relative realistic prediction. It can also be noticed that when deep learning models take the three-level echoes as input, the evaluation scores on CSI and HSS improved and FAR reduced. Overall, our model is comprehensively the best one, both in echo motion and evolution prediction, visual realistic reliability, and quantitative evaluation.

### 3.5. Performance Analysis

In this section, we have evaluated the performance on time-consuming and memory-consuming for MLC-LSTM, ConvLSTM, TrajGRU, TREC, and Optical flow. The results are shown in Table 7. The TREC and Optical flow consume the least memory, but they need about 1 to 2 s to extrapolate one sample. The deep learning models usually request larger memory and take a while for model training, but once the training is finished, the converged model can be near-instantaneous. For our model, training the MLC-LSTM per iteration in our hardware conditions takes about 0.56 s, and the full training procedure usually lasts 3 to 6 h, but for the test, it only needs 0.0927s to extrapolate one sample, which can satisfy the real-time application requirement. For memory consumption, although it occupies 6985.04 MB video memory during the training phase, it is less than 8 GB. Therefore, our model can be trained and deployed conveniently conduct on any GPU which memory is equal to or greater than 8 GB.

## 4. Conclusions

In this paper, we have studied the weather radar extrapolation for short-term weather forecasting and precipitation nowcasting, which is the prediction of the appearance, intensity, and distribution of future echoes according to historical echo observations. Although the recent applications of deep learning for extrapolation have made remarkable progress compared with the traditional extrapolation methods, there still exist two major problems. The first one is that the echo evolution has been little investigated which also influences the accuracy of extrapolation. The second is that current deep learning models generate blurry predictions as the extrapolation goes deeper. To address the two issues, first, we proposed the MLC-LSTM for exploiting the spatiotemporal correlation between multi-level radar echoes and modeling echo evolution. Then we adopted adversarial training to make the extrapolated echo realistic and sharp.

To train and test our model, a real-life multi-level radar echoes dataset was built. Through the extrapolation experiments, it demonstrated that our model can effectively predict the echo motion and evolution while the blurry prediction problem is avoided, the extrapolated echo is visually realistic and fine-grained. For quantitative evaluation, our model also achieved a comprehensive optimal score on metrics that are commonly used for precipitation nowcasting. In terms of hardware performance, our model can be easily and cost-effectively employed on most common hardware setups. Its running speed also meets the requirement of the application. Therefore, our model has promising potential for actual short-term weather forecasting practice.

In addition to the advantages of our model, there is still room for improvement. First, even though the echo motion and evolution have been modeled appropriately, the extrapolated echo shape did not match the ground-truth perfectly, and sometimes the echo intensity fluctuated, and the consistency of intensity was not guaranteed. For this, we consider that introducing some kind of morphometric loss and the technique for maintaining intensity consistency are useful. Second, in reality, short-term weather forecasting practice requires the quality of extrapolation to remain reliable, even when the validation time is greater than 2 h (more than 20 echo frames). Therefore, a well-designed long-term extrapolation model would be necessary, which can ensure both the long-term extrapolation accuracy and model performance. The above two problems will be studied in our future work.

## Figures and Tables

**Table 1:** The ‘In Size’, ‘Ch Num’, ‘Kernel’ and ‘stride’ are each layer’s input size, number of channels, convolution (deconvolution) kernel size and convolution (deconvolution) stride, respectively. (**a**) The parameters configuration of the encoder; (**b**) The parameters configuration of the predictor.

(**a**)				
**Encoder**	**In Size**	**Ch Num**	**Kernel**	**Stride**
Fusion1/Conv1	128 × 128	64	5 × 5	1 × 1
Fusion1/Conv2	128 × 128	64	3 × 3	2 × 2
ConvLSTM1	64 × 64	64	3 × 3	1 × 1
Fusion2/Conv2	64 × 64	128	3 × 3	2 × 2
ConvLSTM3	32 × 32	128	3 × 3	1 × 1
(**b**)				
**Predictor**	**In Size**	**Ch Num**	**Kernel**	**Stride**
ConvLSTM4	32 × 32	128	3 × 3	1 × 1
Separation2/DeConv1	32 × 32	64	3 × 3	2 × 2
ConvLSTM2	64 × 64	64	3 × 3	1 × 1
Separation1/DeConv1	64 × 64	64	3 × 3	2 × 2

**Table 2:** Quantitative evaluation results of three models on six metrics. The mean value of six metrics of all 15 extrapolation steps is reported. The figures with bold-face indicate the best while the figures with underline indicate the second.

Model	POD	FAR	CSI	HSS	SSIM	PSNR
MLC-LSTM(Ours)	0.6538	**0.2818**	**0.5348**	**0.6298**	**0.6755**	**22.7943**
MLC-LSTM(1 layer)	**0.7035**	0.3496	0.5230	0.6143	0.6353	22.2052
MLC-LSTM(3 layers)	0.6513	0.2963	0.5260	0.6195	0.6598	22.4943

**Table 3:** Quantitative evaluation results of three models on six metrics. The mean value of six metrics of all 15 extrapolation steps is reported. The figures with bold-face indicate the best while the figures with underline indicate the second.

Model	POD	FAR	CSI	HSS	SSIM	PSNR
MLC-LSTM(Ours)	0.6538	**0.2818**	**0.5348**	**0.6298**	**0.6755**	**22.7943**
MLC-LSTM(w/o dropout)	**0.6750**	0.3332	0.5227	0.6149	0.6271	22.0239
MLC-LSTM(0.5 dropout)	0.6306	0.2962	0.5146	0.6085	0.6619	22.3699

**Table 4:** Quantitative evaluation results on six metrics of Multi-Level Correlation Long Short-Term Memory (MLC-LSTM) trained with different loss schemes.

Model	POD	FAR	CSI	HSS	SSIM	PSNR
MLC-LSTM(Ours)	0.6538	0.2818	**0.5348**	**0.6298**	**0.6755**	22.7943
MLC-LSTM(MAE + adv)	0.6499	0.3040	0.5233	0.6167	0.6627	22.3537
MLC-LSTM(MSE + adv)	**0.6608**	0.3281	0.5169	0.6091	0.6391	22.3504
MLC-LSTM(adv)	0.4700	0.3774	0.3818	0.4601	0.5631	21.3080
MLC-LSTM(MSE + MAE)	0.6012	**0.2295**	0.5163	0.6090	0.6729	**22.8769**

**Table 5:** Quantitative evaluation results on six metrics of MLC-LSTM trained with different updating ratios.

Model	POD	FAR	CSI	HSS	SSIM	PSNR
MLC-LSTM(Ours)	0.6538	**0.2818**	**0.5348**	**0.6298**	**0.6755**	**22.7943**
MLC-LSTM(1G:2D)	**0.7029**	0.4344	0.4673	0.5509	0.6052	20.9115
MLC-LSTM(1G:1D)	0.6524	0.3391	0.5058	0.5977	0.6373	21.7172
MLC-LSTM(3G:1D)	0.6414	0.2845	0.5260	0.6212	0.6735	22.5444

**Table 6:** Quantitative evaluation results of seven models on six metrics. The mean value of six metrics of 5, 10, and 15 extrapolation steps is reported, respectively (corresponding to 0.5, 1, and 1.5 h). (**a**) Evaluation results on the probability of detection (POD) and false alarm rate (FAR); (**b**) Evaluation results on the critical success index (CSI) and Heidke skill score (HSS); (**c**) Evaluation results on the structural similarity index measure (SSIM) and peak signal to noise ratio (PSNR).

(**a**)						
**Model**	**POD**			**FAR**		
	**0.5h**	**1h**	**1.5h**	**0.5h**	**1h**	**1.5h**
MLC-LSTM(Ours)	**0.7373**	**0.6937**	0.6538	0.1868	0.2404	0.2818
MLC-LSTM(one-level input)	0.7354	0.6934	**0.6564**	0.2179	0.2739	0.3203
ConvLSTM	0.7176	0.6381	0.5793	0.2015	0.2544	0.2946
ConvLSTM(three-level input)	0.7168	0.6452	0.5935	0.1867	0.2437	0.2871
TrajGRU	0.6967	0.6333	0.5813	**0.1788**	**0.2108**	**0.2387**
TREC	0.5545	0.4755	0.4249	0.3779	0.4574	0.5053
Optical flow	0.5491	0.4579	0.4024	0.4088	0.4910	0.5438
(**b**)						
**Model**	**CSI**			**HSS**		
	**0.5h**	**1h**	**1.5h**	**0.5h**	**1h**	**1.5h**
MLC-LSTM(Ours)	**0.6396**	**0.5810**	**0.5348**	**0.7346**	**0.6779**	**0.6298**
MLC-LSTM(one-level input)	0.6213	0.5636	0.5179	0.7171	0.6599	0.6111
ConvLSTM	0.6177	0.5382	0.4819	0.7118	0.6301	0.5681
ConvLSTM(three-level input)	0.6258	0.5486	0.4949	0.7198	0.6414	0.5827
TrajGRU	0.6091	0.5466	0.4971	0.7055	0.6421	0.5879
TREC	0.4257	0.3514	0.3079	0.5144	0.4237	0.3676
Optical flow	0.4172	0.3367	0.2898	0.4984	0.3980	0.3359
(**c**)						
**Model**	**SSIM**			**PSNR**		
	**0.5h**	**1h**	**1.5h**	**0.5h**	**1h**	**1.5h**
MLC-LSTM(Ours)	**0.7495**	**0.7037**	**0.6755**	24.5140	**23.4815**	22.7943
MLC-LSTM(one-level input)	0..7346	0.6876	0.6553	23.7549	22.7504	22.0705
ConvLSTM	0.7371	0.6888	0.6626	24.4339	23.2579	22.5643
ConvLSTM(three-level input)	0.7413	0.6920	0.6644	**24.6442**	23.4424	22.7297
TrajGRU	0.7256	0.6901	0.6685	24.3465	23.4219	**22.8305**
TREC	0.5973	0.5466	0.5215	19.0707	18.3897	18.0853
Optical flow	0.5763	0.5337	0.5104	19.5417	18.4640	17.9306

**Table 7:** Performance evaluation results of the MLC-SLTM, ConvLSTM, TrajGRU, TREC, and Optical flow.

Model	Training Time (s/iteration)	Test Time (s/sample)	Model Parameters	Memory Consumption (MB)
MLC-LSTM	0.56	0.0927	4191874	6985.04
ConvLSTM	0.44	0.1376	891521	3541.04
TrajGRU	0.68	0.1124	12398497	4085.76
TREC	-	1.5197	-	131.25
Optical flow	-	2.3848	-	134.40
